# Tetra­chlorido(1,10-phenanthroline-κ^2^ *N*,*N*′)platinum(IV) acetonitrile hemisolvate

**DOI:** 10.1107/S1600536809002359

**Published:** 2009-01-23

**Authors:** Nam-Ho Kim, In-Chul Hwang, Kwang Ha

**Affiliations:** a School of Applied Chemical Engineering, Research Institute of Catalysis, Chonnam National University, Gwangju 500-757, Republic of Korea; b Department of Chemistry, Pohang University of Science and Technology, Pohang 790-784, Republic of Korea

## Abstract

The asymmetric unit of the title compound, [PtCl_4_(C_12_H_8_N_2_)]·0.5CH_3_CN, contains two crystallographically independent Pt^IV^ complexes with very similar geometry and one solvent mol­ecule. In the complexes, each Pt^IV^ ion is six-coordinated in a distorted octa­hedral environment by two N atoms of the 1,10-phenanthroline ligand and four Cl atoms. Because of the different *trans* effects of the N and Cl atoms, the Pt—Cl bonds *trans* to the N atom are slightly shorter than those *trans* to the Cl atom. The compound displays numerous inter­molecular π–π inter­actions between six-membered rings, with a shortest centroid-to-centroid distance of 3.654 Å. There are also weak intra- and inter­molecular C—H⋯Cl hydrogen bonds.

## Related literature

For details of some other Pt–phenanthroline complexes, see: Buse *et al.* (1977 ▶); Fanizzi *et al.* (1996 ▶). For related Pt–bipyridine complexes, see: Hambley (1986 ▶); Hojjat Kashani *et al.* (2008 ▶).
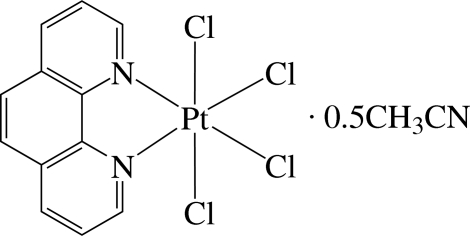


## Experimental

### 

#### Crystal data

[PtCl_4_(C_12_H_8_N_2_)]·0.5C_2_H_3_N
*M* *_r_* = 1075.24
Triclinic, 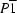

*a* = 7.671 (5) Å
*b* = 12.619 (8) Å
*c* = 16.63 (1) Å
α = 89.70 (1)°
β = 87.46 (1)°
γ = 78.797 (7)°
*V* = 1577 (2) Å^3^
*Z* = 2
Mo *K*α radiation
μ = 9.56 mm^−1^
*T* = 293 (2) K
0.55 × 0.30 × 0.30 mm

#### Data collection

Bruker SMART 1000 CCD diffractometer
Absorption correction: multi-scan (*SADABS*; Bruker, 2000 ▶) *T*_min_ = 0.035, *T*_max_ = 0.057
8700 measured reflections
5856 independent reflections
5250 reflections with *I* > 2σ(*I*)
*R*_int_ = 0.027

#### Refinement

*R*[*F* ^2^ > 2σ(*F* ^2^)] = 0.035
*wR*(*F* ^2^) = 0.104
*S* = 1.09
5856 reflections
372 parameters
H-atom parameters constrained
Δρ_max_ = 1.97 e Å^−3^
Δρ_min_ = −2.05 e Å^−3^

### 

Data collection: *SMART* (Bruker, 2000 ▶); cell refinement: *SAINT* (Bruker, 2000 ▶); data reduction: *SAINT*; program(s) used to solve structure: *SHELXS97* (Sheldrick, 2008 ▶); program(s) used to refine structure: *SHELXL97* (Sheldrick, 2008 ▶); molecular graphics: *ORTEP-3* (Farrugia, 1997 ▶) and *PLATON* (Spek, 2003 ▶); software used to prepare material for publication: *SHELXL97*.

## Supplementary Material

Crystal structure: contains datablocks global, I. DOI: 10.1107/S1600536809002359/im2096sup1.cif

Structure factors: contains datablocks I. DOI: 10.1107/S1600536809002359/im2096Isup2.hkl

Additional supplementary materials:  crystallographic information; 3D view; checkCIF report

## Figures and Tables

**Table 1:** Hydrogen-bond geometry (Å, °)

*D*—H⋯*A*	*D*—H	H⋯*A*	*D*⋯*A*	*D*—H⋯*A*
C1—H1⋯Cl2	0.93	2.68	3.243 (7)	120
C1—H1⋯Cl6	0.93	2.75	3.632 (7)	158
C6—H6⋯Cl8^i^	0.93	2.74	3.637 (8)	163
C10—H10⋯Cl1	0.93	2.72	3.275 (9)	120
C13—H13⋯Cl6	0.93	2.68	3.248 (9)	120
C15—H15⋯Cl1^ii^	0.93	2.79	3.669 (9)	159
C21—H21⋯Cl2^iii^	0.93	2.72	3.451 (9)	136
C22—H22⋯Cl5	0.93	2.74	3.297 (9)	120

Symmetry codes: (i) 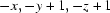; (ii) 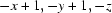; (iii) 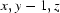.

**Fig. 1.**
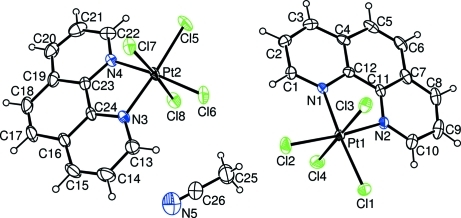
The structure of the title compound, with displacement ellipsoids drawn at the 30% probability level for non-H atoms.

**Fig. 2.**
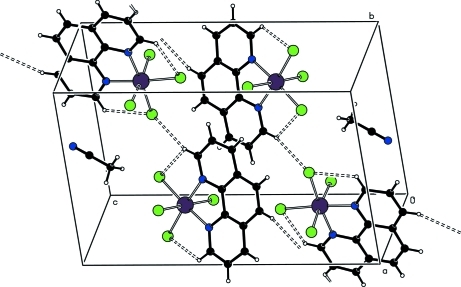
View of the unit-cell contents of the title compound. Hydrogen-bond interactions are drawn with dashed lines.

## supplementary crystallographic information

### Comment

The asymmetric unit of the title compound, [PtCl_4_(C_12_H_8_N_2_)].0.5CH_3_CN, contains two crystallographically independent Pt^IV^ complexes with identical geometry and a CH_3_CN solvent molecule (Fig. 1 and 2). In the complexes, each Pt^4+^ ion is six-coordinated in a distorted octahedral environment by two N atoms of the 1,10-phenanthroline ligand and four Cl atoms. The main contributions to the distortion are the tight N—Pt—N chelate angles (82.0 (2)° and 80.9 (2)°), which result in non-linear *trans* axes (Cl—Pt—N = 174.7 (2)–175.7 (2)°, Cl—Pt—Cl = 177.57 (8)° and 175.68 (7)°). Because of the different *trans* effects of the N and Cl atoms, the Pt—Cl bonds *trans* to the N atom (lengths: 2.294 (2), 2.297 (2), 2.301 (2) and 2.298 (2) Å; mean length: 2.298 (2) Å) are slightly shorter than bond lengths to mutually *trans* Cl atoms (lengths: 2.322 (2), 2.312 (2), 2.302 (2) and 2.309 (2) Å; mean length: 2.311 (2) Å). The compound displays numerous intermolecular π–π interactions between six-membered rings, with a shortest centroid–centroid distance of 3.654 Å. There are also weak intra- and intermolecular C—H···Cl hydrogen bonds (Table 1).

### Experimental

To a solution of K_2_PtCl_6_ (0.2026 g, 0.417 mmol) in H_2_O (10 ml) was added 1,10-phenanthroline (0.2162 g, 1.200 mmol) in MeOH (10 ml), and stirred for 5 h at room temperature. The formed precipitate was separated by filtration and washed with water and MeOH and dried under vacuum, to give a yellow powder (0.1710 g). Crystals suitable for X-ray analysis were obtained by slow evaporation from a CH_3_CN solution.

### Refinement

H atoms were positioned geometrically and allowed to ride on their respective parent atoms [C—H = 0.93 (aromatic) or 0.96 Å (CH_3_) and *U*_iso_(H) = 1.2*U*_eq_(C) or 1.5*U*_eq_(methyl C)].

### Crystal data

**Table:**

[PtCl_4_(C_12_H_8_N_2_)]·0.5C_2_H_3_N	*Z* = 2
*M**_r_* = 1075.24	*F*(000) = 1004
Triclinic, *P*1	*D*_x_ = 2.264 Mg m^−^^3^
Hall symbol: -P 1	Mo *K*α radiation, λ = 0.71073 Å
*a* = 7.671 (5) Å	Cell parameters from 907 reflections
*b* = 12.619 (8) Å	θ = 3.0–26.4°
*c* = 16.63 (1) Å	µ = 9.56 mm^−^^1^
α = 89.70 (1)°	*T* = 293 K
β = 87.46 (1)°	Rod, yellow
γ = 78.797 (7)°	0.55 × 0.30 × 0.30 mm
*V* = 1577 (2) Å^3^	

### Data collection

**Table:**

Bruker SMART 1000 CCD diffractometer	5856 independent reflections
Radiation source: fine-focus sealed tube	5250 reflections with *I* > 2σ(*I*)
graphite	*R*_int_ = 0.027
φ and ω scans	θ_max_ = 25.7°, θ_min_ = 1.2°
Absorption correction: multi-scan (*SADABS*; Bruker, 2000)	*h* = −9→9
*T*_min_ = 0.035, *T*_max_ = 0.057	*k* = −15→14
8700 measured reflections	*l* = −9→20

### Refinement

**Table:**

Refinement on *F*^2^	Secondary atom site location: difference Fourier map
Least-squares matrix: full	Hydrogen site location: inferred from neighbouring sites
*R*[*F*^2^ > 2σ(*F*^2^)] = 0.035	H-atom parameters constrained
*wR*(*F*^2^) = 0.104	*w* = 1/[σ^2^(*F*_o_^2^) + (0.0696*P*)^2^ + 1.1983*P*] where *P* = (*F*_o_^2^ + 2*F*_c_^2^)/3
*S* = 1.09	(Δ/σ)_max_ = 0.001
5856 reflections	Δρ_max_ = 1.97 e Å^−^^3^
372 parameters	Δρ_min_ = −2.05 e Å^−^^3^
0 restraints	Extinction correction: *SHELXL97* (Sheldrick, 2008), Fc^*^=kFc[1+0.001xFc^2^λ^3^/sin(2θ)]^-1/4^
Primary atom site location: structure-invariant direct methods	Extinction coefficient: 0.0069 (3)

### Special details

**Table:**

Geometry. All e.s.d.'s (except the e.s.d. in the dihedral angle between two l.s. planes) are estimated using the full covariance matrix. The cell e.s.d.'s are taken into account individually in the estimation of e.s.d.'s in distances, angles and torsion angles; correlations between e.s.d.'s in cell parameters are only used when they are defined by crystal symmetry. An approximate (isotropic) treatment of cell e.s.d.'s is used for estimating e.s.d.'s involving l.s. planes.
Refinement. Refinement of *F*^2^ against ALL reflections. The weighted *R*-factor *wR* and goodness of fit *S* are based on *F*^2^, conventional *R*-factors *R* are based on *F*, with *F* set to zero for negative *F*^2^. The threshold expression of *F*^2^ > σ(*F*^2^) is used only for calculating *R*-factors(gt) *etc*. and is not relevant to the choice of reflections for refinement. *R*-factors based on *F*^2^ are statistically about twice as large as those based on *F*, and *R*- factors based on ALL data will be even larger.

### Fractional atomic coordinates and isotropic or equivalent isotropic displacement parameters (Å^2^)

**Table:**

	*x*	*y*	*z*	*U*_iso_*/*U*_eq_	
Pt1	0.22405 (3)	0.743662 (18)	0.348926 (14)	0.03174 (12)	
Cl1	0.1723 (3)	0.90349 (15)	0.27957 (12)	0.0592 (6)	
Cl2	0.4276 (3)	0.66204 (15)	0.25175 (12)	0.0586 (6)	
Cl3	0.4436 (2)	0.80367 (14)	0.41769 (12)	0.0440 (4)	
Cl4	−0.0011 (3)	0.68354 (17)	0.28563 (13)	0.0604 (6)	
N1	0.2550 (6)	0.6080 (4)	0.4184 (3)	0.0259 (10)	
N2	0.0449 (7)	0.8027 (4)	0.4402 (3)	0.0329 (12)	
C1	0.3525 (9)	0.5108 (5)	0.4027 (4)	0.0336 (14)	
H1	0.4154	0.4982	0.3534	0.040*	
C2	0.3624 (9)	0.4275 (5)	0.4583 (4)	0.0379 (15)	
H2	0.4316	0.3602	0.4456	0.046*	
C3	0.2724 (9)	0.4430 (6)	0.5313 (5)	0.0406 (17)	
H3	0.2806	0.3871	0.5685	0.049*	
C4	0.1658 (9)	0.5460 (5)	0.5493 (4)	0.0336 (14)	
C5	0.0584 (10)	0.5727 (7)	0.6212 (4)	0.0442 (18)	
H5	0.0636	0.5217	0.6621	0.053*	
C6	−0.0497 (10)	0.6690 (7)	0.6318 (4)	0.0466 (18)	
H6	−0.1184	0.6834	0.6794	0.056*	
C7	−0.0609 (9)	0.7500 (6)	0.5710 (4)	0.0408 (16)	
C8	−0.1779 (10)	0.8520 (7)	0.5756 (6)	0.053 (2)	
H8	−0.2517	0.8709	0.6213	0.064*	
C9	−0.1814 (10)	0.9223 (7)	0.5128 (6)	0.056 (2)	
H9	−0.2594	0.9887	0.5151	0.067*	
C10	−0.0692 (10)	0.8948 (6)	0.4460 (5)	0.0457 (18)	
H10	−0.0745	0.9432	0.4034	0.055*	
C11	0.0471 (8)	0.7290 (5)	0.5010 (4)	0.0321 (14)	
C12	0.1595 (8)	0.6274 (5)	0.4905 (4)	0.0272 (12)	
Pt2	0.66093 (3)	0.20111 (2)	0.213982 (14)	0.03046 (12)	
Cl5	0.6389 (3)	0.17427 (19)	0.35077 (11)	0.0554 (5)	
Cl6	0.6342 (3)	0.38351 (16)	0.23497 (13)	0.0553 (5)	
Cl7	0.9662 (2)	0.17316 (15)	0.21686 (12)	0.0454 (4)	
Cl8	0.3574 (2)	0.21957 (18)	0.20343 (12)	0.0523 (5)	
N3	0.6888 (7)	0.2128 (4)	0.0925 (3)	0.0320 (12)	
N4	0.6814 (7)	0.0429 (4)	0.1845 (3)	0.0317 (12)	
C13	0.6842 (12)	0.3021 (7)	0.0489 (5)	0.052 (2)	
H13	0.6623	0.3694	0.0740	0.063*	
C14	0.7131 (14)	0.2939 (9)	−0.0360 (5)	0.068 (3)	
H14	0.7111	0.3558	−0.0668	0.081*	
C15	0.7435 (13)	0.1957 (8)	−0.0718 (5)	0.061 (2)	
H15	0.7643	0.1910	−0.1273	0.073*	
C16	0.7445 (10)	0.1033 (6)	−0.0286 (4)	0.0454 (17)	
C17	0.7660 (13)	−0.0050 (8)	−0.0609 (5)	0.065 (3)	
H17	0.7849	−0.0152	−0.1162	0.078*	
C18	0.7596 (13)	−0.0894 (8)	−0.0150 (6)	0.070 (3)	
H18	0.7768	−0.1575	−0.0386	0.084*	
C19	0.7265 (10)	−0.0793 (6)	0.0715 (5)	0.0491 (19)	
C20	0.7087 (12)	−0.1636 (7)	0.1247 (7)	0.060 (2)	
H20	0.7216	−0.2339	0.1054	0.073*	
C21	0.6734 (12)	−0.1430 (7)	0.2032 (7)	0.064 (3)	
H21	0.6576	−0.1989	0.2377	0.077*	
C22	0.6599 (10)	−0.0387 (6)	0.2342 (5)	0.0478 (19)	
H22	0.6362	−0.0258	0.2890	0.057*	
C23	0.7100 (8)	0.0249 (5)	0.1044 (4)	0.0338 (14)	
C24	0.7161 (8)	0.1149 (5)	0.0555 (4)	0.0340 (14)	
N5	0.225 (2)	0.4009 (12)	0.0069 (10)	0.153 (6)	
C25	0.0741 (19)	0.4592 (9)	0.1403 (7)	0.094 (4)	
H26A	0.1404	0.5048	0.1669	0.140*	
H26B	0.0692	0.3965	0.1726	0.140*	
H26C	−0.0445	0.4984	0.1328	0.140*	
C26	0.159 (2)	0.4266 (10)	0.0640 (8)	0.098 (4)	

### Atomic displacement parameters (Å^2^)

**Table:**

	*U*^11^	*U*^22^	*U*^33^	*U*^12^	*U*^13^	*U*^23^
Pt1	0.04667 (19)	0.02322 (16)	0.02332 (16)	−0.00292 (11)	0.00364 (11)	−0.00038 (10)
Cl1	0.1033 (17)	0.0321 (9)	0.0373 (10)	−0.0022 (9)	0.0031 (11)	0.0082 (8)
Cl2	0.0919 (15)	0.0362 (9)	0.0423 (11)	−0.0070 (9)	0.0334 (11)	−0.0047 (8)
Cl3	0.0463 (9)	0.0337 (8)	0.0532 (11)	−0.0113 (7)	−0.0004 (8)	−0.0019 (8)
Cl4	0.0792 (14)	0.0493 (11)	0.0543 (12)	−0.0101 (10)	−0.0322 (11)	−0.0024 (9)
N1	0.030 (3)	0.028 (3)	0.021 (2)	−0.008 (2)	−0.002 (2)	−0.001 (2)
N2	0.036 (3)	0.030 (3)	0.030 (3)	−0.001 (2)	0.000 (2)	−0.004 (2)
C1	0.036 (3)	0.030 (3)	0.035 (4)	−0.007 (3)	0.001 (3)	−0.005 (3)
C2	0.039 (4)	0.031 (3)	0.045 (4)	−0.010 (3)	−0.009 (3)	0.001 (3)
C3	0.040 (4)	0.039 (4)	0.048 (4)	−0.017 (3)	−0.017 (3)	0.014 (3)
C4	0.036 (3)	0.044 (4)	0.027 (3)	−0.020 (3)	−0.005 (3)	0.000 (3)
C5	0.055 (4)	0.062 (5)	0.024 (3)	−0.034 (4)	−0.004 (3)	0.001 (3)
C6	0.051 (4)	0.063 (5)	0.032 (4)	−0.029 (4)	0.009 (3)	−0.010 (3)
C7	0.034 (3)	0.058 (4)	0.035 (4)	−0.019 (3)	0.005 (3)	−0.015 (3)
C8	0.038 (4)	0.060 (5)	0.060 (5)	−0.010 (3)	0.013 (4)	−0.033 (4)
C9	0.042 (4)	0.039 (4)	0.085 (7)	−0.005 (3)	0.011 (4)	−0.026 (4)
C10	0.046 (4)	0.036 (4)	0.053 (5)	−0.001 (3)	−0.003 (4)	−0.014 (3)
C11	0.026 (3)	0.040 (3)	0.031 (3)	−0.008 (3)	0.001 (3)	−0.011 (3)
C12	0.029 (3)	0.031 (3)	0.024 (3)	−0.010 (2)	−0.005 (2)	0.004 (2)
Pt2	0.02315 (16)	0.04333 (18)	0.02481 (16)	−0.00705 (10)	0.00409 (10)	−0.01164 (11)
Cl5	0.0534 (11)	0.0889 (15)	0.0243 (8)	−0.0158 (10)	0.0042 (8)	−0.0096 (9)
Cl6	0.0596 (12)	0.0453 (10)	0.0580 (12)	−0.0041 (8)	0.0057 (10)	−0.0247 (9)
Cl7	0.0250 (8)	0.0594 (11)	0.0528 (11)	−0.0107 (7)	0.0002 (7)	−0.0201 (9)
Cl8	0.0228 (8)	0.0838 (14)	0.0487 (11)	−0.0072 (8)	0.0031 (7)	−0.0119 (10)
N3	0.031 (3)	0.039 (3)	0.028 (3)	−0.011 (2)	0.005 (2)	−0.006 (2)
N4	0.027 (3)	0.038 (3)	0.033 (3)	−0.013 (2)	0.001 (2)	−0.007 (2)
C13	0.068 (5)	0.044 (4)	0.046 (5)	−0.014 (4)	0.003 (4)	0.001 (4)
C14	0.089 (7)	0.075 (6)	0.042 (5)	−0.024 (5)	0.004 (5)	0.018 (5)
C15	0.080 (6)	0.075 (6)	0.025 (4)	−0.010 (5)	0.002 (4)	−0.002 (4)
C16	0.048 (4)	0.053 (4)	0.033 (4)	−0.004 (3)	0.002 (3)	−0.013 (3)
C17	0.073 (6)	0.076 (6)	0.036 (4)	0.007 (5)	−0.002 (4)	−0.024 (4)
C18	0.078 (6)	0.061 (6)	0.064 (6)	0.008 (5)	−0.016 (5)	−0.038 (5)
C19	0.046 (4)	0.042 (4)	0.059 (5)	−0.005 (3)	−0.008 (4)	−0.016 (4)
C20	0.059 (5)	0.038 (4)	0.084 (7)	−0.006 (4)	−0.018 (5)	−0.012 (4)
C21	0.063 (6)	0.047 (5)	0.086 (7)	−0.016 (4)	−0.024 (5)	0.015 (5)
C22	0.040 (4)	0.056 (5)	0.052 (5)	−0.018 (3)	−0.007 (4)	0.015 (4)
C23	0.027 (3)	0.039 (4)	0.035 (4)	−0.005 (3)	0.000 (3)	−0.013 (3)
C24	0.029 (3)	0.043 (4)	0.029 (3)	−0.004 (3)	0.002 (3)	−0.012 (3)
N5	0.179 (15)	0.133 (12)	0.121 (12)	0.031 (10)	0.022 (11)	−0.030 (10)
C25	0.127 (10)	0.069 (7)	0.081 (8)	−0.015 (7)	0.027 (8)	−0.008 (6)
C26	0.131 (12)	0.073 (8)	0.075 (8)	0.017 (7)	−0.010 (8)	−0.020 (7)

### Geometric parameters (Å, °)

**Table:**

Pt1—N1	2.041 (5)	Pt2—Cl5	2.301 (2)
Pt1—N2	2.044 (5)	Pt2—Cl7	2.302 (2)
Pt1—Cl1	2.294 (2)	Pt2—Cl8	2.309 (2)
Pt1—Cl2	2.297 (2)	N3—C13	1.332 (10)
Pt1—Cl4	2.312 (2)	N3—C24	1.358 (8)
Pt1—Cl3	2.322 (2)	N4—C22	1.348 (9)
N1—C1	1.327 (8)	N4—C23	1.353 (8)
N1—C12	1.374 (8)	C13—C14	1.421 (12)
N2—C10	1.313 (9)	C13—H13	0.9300
N2—C11	1.369 (9)	C14—C15	1.351 (14)
C1—C2	1.390 (10)	C14—H14	0.9300
C1—H1	0.9300	C15—C16	1.365 (12)
C2—C3	1.365 (11)	C15—H15	0.9300
C2—H2	0.9300	C16—C24	1.410 (10)
C3—C4	1.420 (10)	C16—C17	1.448 (12)
C3—H3	0.9300	C17—C18	1.315 (14)
C4—C12	1.411 (9)	C17—H17	0.9300
C4—C5	1.425 (10)	C18—C19	1.451 (13)
C5—C6	1.339 (11)	C18—H18	0.9300
C5—H5	0.9300	C19—C20	1.404 (13)
C6—C7	1.427 (11)	C19—C23	1.408 (10)
C6—H6	0.9300	C20—C21	1.336 (14)
C7—C11	1.393 (9)	C20—H20	0.9300
C7—C8	1.420 (11)	C21—C22	1.400 (12)
C8—C9	1.364 (13)	C21—H21	0.9300
C8—H8	0.9300	C22—H22	0.9300
C9—C10	1.378 (12)	C23—C24	1.400 (10)
C9—H9	0.9300	N5—C26	1.077 (17)
C10—H10	0.9300	C25—C26	1.425 (18)
C11—C12	1.407 (9)	C25—H26A	0.9600
Pt2—N3	2.030 (5)	C25—H26B	0.9600
Pt2—N4	2.032 (5)	C25—H26C	0.9600
Pt2—Cl6	2.298 (2)		
			
N1—Pt1—N2	82.0 (2)	N4—Pt2—Cl5	95.07 (17)
N1—Pt1—Cl1	174.98 (15)	Cl6—Pt2—Cl5	90.10 (8)
N2—Pt1—Cl1	92.96 (16)	N3—Pt2—Cl7	87.56 (15)
N1—Pt1—Cl2	92.78 (15)	N4—Pt2—Cl7	89.02 (15)
N2—Pt1—Cl2	174.77 (15)	Cl6—Pt2—Cl7	91.93 (7)
Cl1—Pt1—Cl2	92.23 (8)	Cl5—Pt2—Cl7	90.84 (7)
N1—Pt1—Cl4	88.58 (15)	N3—Pt2—Cl8	89.53 (15)
N2—Pt1—Cl4	88.78 (17)	N4—Pt2—Cl8	87.37 (15)
Cl1—Pt1—Cl4	91.60 (9)	Cl6—Pt2—Cl8	91.46 (8)
Cl2—Pt1—Cl4	90.38 (10)	Cl5—Pt2—Cl8	91.84 (7)
N1—Pt1—Cl3	90.10 (15)	Cl7—Pt2—Cl8	175.68 (7)
N2—Pt1—Cl3	89.02 (17)	C13—N3—C24	120.0 (6)
Cl1—Pt1—Cl3	89.54 (8)	C13—N3—Pt2	127.6 (5)
Cl2—Pt1—Cl3	91.72 (9)	C24—N3—Pt2	112.3 (4)
Cl4—Pt1—Cl3	177.57 (8)	C22—N4—C23	120.1 (6)
C1—N1—C12	120.0 (5)	C22—N4—Pt2	127.4 (5)
C1—N1—Pt1	128.9 (4)	C23—N4—Pt2	112.4 (4)
C12—N1—Pt1	111.2 (4)	N3—C13—C14	119.6 (8)
C10—N2—C11	118.9 (6)	N3—C13—H13	120.2
C10—N2—Pt1	129.9 (5)	C14—C13—H13	120.2
C11—N2—Pt1	111.1 (4)	C15—C14—C13	119.7 (9)
N1—C1—C2	121.3 (6)	C15—C14—H14	120.2
N1—C1—H1	119.4	C13—C14—H14	120.2
C2—C1—H1	119.4	C14—C15—C16	121.8 (8)
C3—C2—C1	121.1 (7)	C14—C15—H15	119.1
C3—C2—H2	119.5	C16—C15—H15	119.1
C1—C2—H2	119.5	C15—C16—C24	116.8 (7)
C2—C3—C4	118.7 (6)	C15—C16—C17	126.4 (8)
C2—C3—H3	120.6	C24—C16—C17	116.8 (7)
C4—C3—H3	120.6	C18—C17—C16	122.4 (8)
C12—C4—C3	117.9 (6)	C18—C17—H17	118.8
C12—C4—C5	117.0 (6)	C16—C17—H17	118.8
C3—C4—C5	125.1 (7)	C17—C18—C19	121.8 (8)
C6—C5—C4	122.1 (7)	C17—C18—H18	119.1
C6—C5—H5	119.0	C19—C18—H18	119.1
C4—C5—H5	119.0	C20—C19—C23	117.4 (8)
C5—C6—C7	120.8 (7)	C20—C19—C18	125.9 (8)
C5—C6—H6	119.6	C23—C19—C18	116.7 (8)
C7—C6—H6	119.6	C21—C20—C19	120.1 (8)
C11—C7—C8	116.4 (7)	C21—C20—H20	120.0
C11—C7—C6	119.1 (7)	C19—C20—H20	120.0
C8—C7—C6	124.5 (7)	C20—C21—C22	121.1 (8)
C9—C8—C7	119.7 (7)	C20—C21—H21	119.5
C9—C8—H8	120.2	C22—C21—H21	119.5
C7—C8—H8	120.2	N4—C22—C21	119.9 (8)
C8—C9—C10	119.9 (7)	N4—C22—H22	120.1
C8—C9—H9	120.0	C21—C22—H22	120.1
C10—C9—H9	120.0	N4—C23—C24	117.1 (6)
N2—C10—C9	122.4 (8)	N4—C23—C19	121.4 (7)
N2—C10—H10	118.8	C24—C23—C19	121.4 (7)
C9—C10—H10	118.8	N3—C24—C23	117.1 (6)
N2—C11—C7	122.6 (6)	N3—C24—C16	122.1 (7)
N2—C11—C12	117.9 (6)	C23—C24—C16	120.8 (6)
C7—C11—C12	119.5 (6)	C26—C25—H26A	109.5
N1—C12—C11	117.4 (5)	C26—C25—H26B	109.5
N1—C12—C4	121.1 (6)	H26A—C25—H26B	109.5
C11—C12—C4	121.4 (6)	C26—C25—H26C	109.5
N3—Pt2—N4	80.9 (2)	H26A—C25—H26C	109.5
N3—Pt2—Cl6	93.96 (16)	H26B—C25—H26C	109.5
N4—Pt2—Cl6	174.73 (16)	N5—C26—C25	178.9 (18)
N3—Pt2—Cl5	175.68 (16)		
			
N2—Pt1—N1—C1	175.1 (6)	N4—Pt2—N3—C13	177.0 (6)
Cl2—Pt1—N1—C1	−4.2 (5)	Cl6—Pt2—N3—C13	−1.8 (6)
Cl4—Pt1—N1—C1	86.1 (5)	Cl7—Pt2—N3—C13	−93.6 (6)
Cl3—Pt1—N1—C1	−95.9 (5)	Cl8—Pt2—N3—C13	89.6 (6)
N2—Pt1—N1—C12	−5.8 (4)	N4—Pt2—N3—C24	−3.5 (4)
Cl2—Pt1—N1—C12	174.9 (4)	Cl6—Pt2—N3—C24	177.7 (4)
Cl4—Pt1—N1—C12	−94.8 (4)	Cl7—Pt2—N3—C24	85.9 (4)
Cl3—Pt1—N1—C12	83.1 (4)	Cl8—Pt2—N3—C24	−90.9 (4)
N1—Pt1—N2—C10	−172.5 (6)	N3—Pt2—N4—C22	−173.2 (6)
Cl1—Pt1—N2—C10	7.8 (6)	Cl5—Pt2—N4—C22	8.3 (5)
Cl4—Pt1—N2—C10	−83.8 (6)	Cl7—Pt2—N4—C22	99.1 (5)
Cl3—Pt1—N2—C10	97.3 (6)	Cl8—Pt2—N4—C22	−83.3 (5)
N1—Pt1—N2—C11	4.8 (4)	N3—Pt2—N4—C23	2.8 (4)
Cl1—Pt1—N2—C11	−174.9 (4)	Cl5—Pt2—N4—C23	−175.7 (4)
Cl4—Pt1—N2—C11	93.5 (4)	Cl7—Pt2—N4—C23	−84.9 (4)
Cl3—Pt1—N2—C11	−85.5 (4)	Cl8—Pt2—N4—C23	92.7 (4)
C12—N1—C1—C2	−0.8 (9)	C24—N3—C13—C14	−1.8 (12)
Pt1—N1—C1—C2	178.1 (5)	Pt2—N3—C13—C14	177.7 (6)
N1—C1—C2—C3	−0.2 (10)	N3—C13—C14—C15	0.5 (15)
C1—C2—C3—C4	0.7 (10)	C13—C14—C15—C16	1.1 (16)
C2—C3—C4—C12	−0.3 (9)	C14—C15—C16—C24	−1.3 (14)
C2—C3—C4—C5	177.1 (6)	C14—C15—C16—C17	176.5 (10)
C12—C4—C5—C6	2.7 (10)	C15—C16—C17—C18	−177.8 (10)
C3—C4—C5—C6	−174.7 (7)	C24—C16—C17—C18	0.0 (13)
C4—C5—C6—C7	−0.5 (11)	C16—C17—C18—C19	1.4 (15)
C5—C6—C7—C11	−2.2 (10)	C17—C18—C19—C20	176.7 (9)
C5—C6—C7—C8	176.9 (7)	C17—C18—C19—C23	−3.0 (13)
C11—C7—C8—C9	1.0 (11)	C23—C19—C20—C21	1.5 (12)
C6—C7—C8—C9	−178.1 (7)	C18—C19—C20—C21	−178.2 (9)
C7—C8—C9—C10	−1.2 (12)	C19—C20—C21—C22	−2.4 (14)
C11—N2—C10—C9	3.1 (11)	C23—N4—C22—C21	2.3 (10)
Pt1—N2—C10—C9	−179.8 (6)	Pt2—N4—C22—C21	178.0 (5)
C8—C9—C10—N2	−0.9 (12)	C20—C21—C22—N4	0.5 (12)
C10—N2—C11—C7	−3.3 (10)	C22—N4—C23—C24	174.7 (6)
Pt1—N2—C11—C7	179.1 (5)	Pt2—N4—C23—C24	−1.6 (7)
C10—N2—C11—C12	174.6 (6)	C22—N4—C23—C19	−3.1 (9)
Pt1—N2—C11—C12	−3.0 (7)	Pt2—N4—C23—C19	−179.4 (5)
C8—C7—C11—N2	1.2 (10)	C20—C19—C23—N4	1.2 (10)
C6—C7—C11—N2	−179.6 (6)	C18—C19—C23—N4	−179.1 (7)
C8—C7—C11—C12	−176.7 (6)	C20—C19—C23—C24	−176.5 (7)
C6—C7—C11—C12	2.5 (9)	C18—C19—C23—C24	3.2 (11)
C1—N1—C12—C11	−174.8 (6)	C13—N3—C24—C23	−176.7 (6)
Pt1—N1—C12—C11	6.1 (7)	Pt2—N3—C24—C23	3.8 (7)
C1—N1—C12—C4	1.3 (9)	C13—N3—C24—C16	1.5 (10)
Pt1—N1—C12—C4	−177.9 (4)	Pt2—N3—C24—C16	−178.0 (5)
N2—C11—C12—N1	−2.1 (8)	N4—C23—C24—N3	−1.5 (9)
C7—C11—C12—N1	175.9 (6)	C19—C23—C24—N3	176.3 (6)
N2—C11—C12—C4	−178.2 (6)	N4—C23—C24—C16	−179.7 (6)
C7—C11—C12—C4	−0.2 (9)	C19—C23—C24—C16	−1.9 (10)
C3—C4—C12—N1	−0.7 (9)	C15—C16—C24—N3	0.0 (11)
C5—C4—C12—N1	−178.3 (5)	C17—C16—C24—N3	−178.0 (7)
C3—C4—C12—C11	175.2 (6)	C15—C16—C24—C23	178.2 (7)
C5—C4—C12—C11	−2.4 (9)	C17—C16—C24—C23	0.2 (10)

### Hydrogen-bond geometry (Å, °)

**Table:**

*D*—H···*A*	*D*—H	H···*A*	*D*···*A*	*D*—H···*A*
C1—H1···Cl2	0.93	2.68	3.243 (7)	120
C1—H1···Cl6	0.93	2.75	3.632 (7)	158
C6—H6···Cl8^i^	0.93	2.74	3.637 (8)	163
C10—H10···Cl1	0.93	2.72	3.275 (9)	120
C13—H13···Cl6	0.93	2.68	3.248 (9)	120
C15—H15···Cl1^ii^	0.93	2.79	3.669 (9)	159
C21—H21···Cl2^iii^	0.93	2.72	3.451 (9)	136
C22—H22···Cl5	0.93	2.74	3.297 (9)	120

Symmetry codes: (i) −*x*, −*y*+1, −*z*+1; (ii) −*x*+1, −*y*+1, −*z*; (iii) *x*, *y*−1, *z*.

## References

[bb1] Bruker (2000). *SADABS*, *SMART* and *SAINT* Bruker AXS Inc., Madison, Wisconsin, USA.

[bb2] Buse, K. D., Keller, H. J. & Pritzkow, H. (1977). *Inorg. Chem.* **16**, 1072–1076.

[bb3] Fanizzi, F. P., Natile, G., Lanfranchi, M., Tiripicchio, A., Laschi, F. & Zanello, P. (1996). *Inorg. Chem.* **35**, 3173–3182. 10.1021/ic960125y 11666514

[bb4] Farrugia, L. J. (1997). *J. Appl. Cryst.* **30**, 565.

[bb5] Hambley, T. W. (1986). *Acta Cryst.* C**42**, 49–51.

[bb6] Hojjat Kashani, L., Amani, V., Yousefi, M. & Khavasi, H. R. (2008). *Acta Cryst.* E**64**, m905–m906. 10.1107/S1600536808016796 PMC2961848 21202769

[bb7] Sheldrick, G. M. (2008). *Acta Cryst.* A**64**, 112–122. 10.1107/S0108767307043930 18156677

[bb8] Spek, A. L. (2003). *J. Appl. Cryst.* **36**, 7–13.

